# Structured reporting of x-ray mammography in the first diagnosis of breast cancer: a Delphi consensus proposal

**DOI:** 10.1007/s11547-022-01478-5

**Published:** 2022-03-18

**Authors:** Emanuele Neri, Vincenza Granata, Stefania Montemezzi, Paolo Belli, Daniela Bernardi, Beniamino Brancato, Francesca Caumo, Massimo Calabrese, Francesca Coppola, Elsa Cossu, Lorenzo Faggioni, Alfonso Frigerio, Roberta Fusco, Antonella Petrillo, Veronica Girardi, Chiara Iacconi, Carolina Marini, Maria Adele Marino, Laura Martincich, Jacopo Nori, Federica Pediconi, Gianni Saguatti, Mario Sansone, Francesco Sardanelli, Gianfranco Paride Scaperrotta, Chiara Zuiani, Eleonora Ciaghi, Marco Montella, Vittorio Miele, Roberto Grassi

**Affiliations:** 1 Department of Translational Research, University of Pisa, Via Roma, 67, 56126 Pisa, Italy; 2 Italian Society of Medical and Interventional Radiology SIRM, SIRM Foundation, Via della Signora 2, 20122 Milan, Italy; 3 Division of Radiology, Istituto Nazionale Tumori IRCCS Fondazione Pascale – IRCCS di Napoli, Naples, Italy; 4 Department of Pathology and Diagnostics, Radiology Unit, Azienda Ospedaliera Universitaria Integrata, P.le Stefani 1, 37126 Verona, Italy; 5 Dipartimento Diagnostica Per Immagini, Fondazione Policlinico Universitario A. Gemelli IRCCS, Radioterapia Oncologica ed Ematologia, Rome, Italy; 6 Breast Imaging and Screening Unit, Department of Radiology, Humanitas Research Hospital, Via Manzoni 56, 20089 Rozzano, Milan Italy; 7 Struttura Complessa Di Senologia Clinica, Istituto Per Lo Studio La Prevenzione E La Rete Oncologica (ISPRO), Via Cosimo il Vecchio, 2, 50139 Florence, Italy; 8 Veneto Institute of Oncology IOV - IRCCS, Padua, Italy; 9 UOC Senologia Diagnostica, IRCCS Ospedale Policlinico San Martino, Genoa, Italy; 10 Division of Radiology, IRCCS Azienda Ospedaliero-Universitaria Di Bologna, Bologna, Italy; 11 Radiology, Department of Emergency, Department of Surgical Science, Tor Vergata University Hospital, University of Rome “Tor Vergata”, Rome, Italy; 12 Breast Cancer Screening Reference Centre, AOU Città Della Salute E Della Scienza, Torino, Italy; 13 Medical Oncology Division, Igea SpA, Naples, Italy; 14 Breast Unit Eusoma Certificated, Department of Breast Imaging and Intervention, Istituto Clinico S. Anna, Via del Franzone 31, 25127 Brescia, Italy; 15 Azienda USL Toscana Nord Ovest (ATNO), Carrara, Italy; 16 S.D.Radiologia Senologica, Azienda Ospedaliero-Universitaria Pisana, Via Roma n.67, 56125 Pisa, Italy; 17 Department of Biomedical Sciences and Morphologic and Functional Imaging, University of Messina, Messina, Italy; 18 Unit of Radiodiagnostics, Ospedale Cardinal G. Massaia -ASL AT, Via Conte Verde 125, 14100 Asti, Italy; 19 Diagnostic Senology Unit, Azienda Ospedaliero-Universitaria Careggi, 50139 Florence, Italy; 20 Department of Radiological, Oncological and Pathological Sciences, Sapienza University of Rome, Rome, Italy; 21 Unit of Senology, Department of Oncology, Bellaria Hospital, Bologna, Italy; 22 Department of Electrical Engineering and Telecommunications, University of Naples “Federico II”, Naples, Italy; 23 Department of Biomedical Sciences for Health, Università Degli Studi Di Milano, Via Luigi Mangiagalli 31, 20133 Milan, Italy; 24 Department of Radiology, Fondazione IRCCS Istituto Nazionale Dei Tumori Di Milano, 20133 Milan, Italy; 25 Institute of Radiology, Department of Medicine, University of Udine, University Hospital “S. Maria Della Misericordia”, P.Le S. Maria Della Misericordia, 15, 33100 Udine, Italy; 26 Healthcare Project Manager: Structured Reporting - Exprivia Spa, Bari, Italy; 27 Department of Mental and Physical Health and Preventive Medicine, University of Campania “Luigi Vanvitelli”, Naples, Italy; 28 Division of Radiology, Azienda Ospedaliera Universitaria Careggi, Florence, Italy; 29 Division of Radiology, Università Degli Studi Della Campania “Luigi Vanvitelli”, Naples, Italy

**Keywords:** Structured Reporting, Breast Cancer, Mammography

## Abstract

**Background:**

Radiology is an essential tool in the management of a patient. The aim of this manuscript was to build structured report (SR) Mammography based in Breast Cancer.

**Methods:**

A working team of 16 experts (group A) was composed to create a SR for Mammography Breast Cancer. A further working group of 4 experts (group B), blinded to the activities of the group A, was composed to assess the quality and clinical usefulness of the SR final draft. Modified Delphi process was used to assess level of agreement for all report sections. Cronbach’s alpha (Cα) correlation coefficient was used to assess internal consistency and to measure quality analysis according to the average inter-item correlation.

**Results:**

The final SR version was built by including *n* = 2 items in Personal Data, *n* = 4 items in Setting, *n* = 2 items in Comparison with previous breast examination, *n* = 19 items in Anamnesis and clinical context; *n* = 10 items in Technique; *n* = 1 item in Radiation dose; *n* = 5 items Parenchymal pattern; *n* = 28 items in Description of the finding; *n* = 12 items in Diagnostic categories and Report and *n* = 1 item in Conclusions. The overall mean score of the experts and the sum of score for structured report were 4.9 and 807 in the second round. The Cronbach’s alpha (Cα) correlation coefficient was 0.82 in the second round. About the quality evaluation, the overall mean score of the experts was 3.3. The Cronbach’s alpha (Cα) correlation coefficient was 0.90.

**Conclusions:**

Structured reporting improves the quality, clarity and reproducibility of reports across departments, cities, countries and internationally and will assist patient management and improve breast health care and facilitate research.

## Introduction

Radiology is an essential tool in the management of a patient. The trend toward personalized imaging-based medicine increasingly requires specialized knowledge in order to be able to answer the particular clinical questions of referring specialists [1–3]. The communication occurs through the report written by the radiologist [4–6]. Describing and comprehending the imaging features as well as disposing for the probability-based differential diagnosis are the radiologist’s principal responsibility [7]. Traditionally, reports are free-form narrative. However, reducing difference in reports and creating guideline-concordant templates is essential to radiology’s success in value-based payment models and suitable for patient care [8, 9]. There has been a strong thrust in recent times on improved structure and standardization in radiology reporting. A notable example to structure in the field of breast imaging where the American College of Radiology (ACR) developed and promulgated the breast imaging reporting and data system (BI-RADS). BI-RADS includes a standardized lexicon for description of breast imaging findings and their clinical management [10].

Several proposal have been supported by the major international societies of radiology for the use of structured reports (SR), as the European Society of Radiology and the Radiological Society of North America in the so called “Structured reporting initiative”. The Italian Society of Medical and Interventional Radiology (SIRM) have made available to members of society several templates that can be used in their daily practice [11].

The advantages of SR derive from several features. In oncological setting, there have been substantial advances in the quality of templates and the statement of imaging features. Several studies were able to show that using a SR caused significant progresses in the clarity and comprehensiveness of imaging findings [12–14]. In a paper on SR during staging phase for pancreatic lesions, the investigated surgeons conveyed that only 25 – 42% of narrative templates described all relevant features for surgical planning while an increase to 69 – 98% was realized in the case of SRs [15].

Outside of oncology, in all radiological fields, SR results in a relevant increase in quality, since, considerably more pertinent information were included in the templates and referring clinicians favored the SR to the free-text report (FTR). SR has advantages that go far beyond communication. In fact, the possibility to archive data concerning contrast medium or radiation exposure with consequent addition to the template would be easy technical employment [16, 17].

Despite all of these promising advances, SR has not yet convert in the clinical practice. A survey of SIRM members noticed that the Italian radiologists know SRs, but only a smaller group habitually use it in clinical practice [18].

Among women, breast cancer is the most commonly diagnosed cancer and the leading cause of cancer-related death in the world [19, 20]. Digital breast tomosynthesis (DBT) has rapidly gained ground in the realm of breast cancer screening and diagnosis [21]. Published data showed the superiority of DBT in comparison with the current standard digital mammography (DM). DBT has been shown to provide improved sensitivity and specificity, as well as improved lesion conspicuity and localization [21]. In this context, it is necessary that the current format of free-text reporting (FTR) should be organized and shifted toward SR. The three main reasons for moving from FTR to SR are quality, datafication quantification and accessibility. A critical quality improvement resulting from the use of SR is standardization. The use of templates in SR provides a checklist as to whether all relevant items for a particular examination have been addressed. Thanks to this “structure”, the radiology report will also allow the association of radiological data and other key clinical features, leading to a precise diagnosis and personalized medicine.

The aim of our study was to propose a structured reporting template for x-ray mammography in the first diagnosis of Breast Cancer, to guide radiologists in a systematic reporting and improve the communication of the report to clinicians.

## Materials and methods

Critical debate between specialist in Breast Radiology based on a multi-round consensus-building modified Delphi method was completed to improve a comprehensive SR for Mammography of patients with Breast Cancer.

### Panel experts

A working group of 16 experts (group A), members of the board of the SIRM study section on breast radiology, was composed to create a structured report for the first diagnosis of breast cancer in x-ray mammography. A further working group of 4 experts in breast radiology (group B), chosen among senior past board members who gave their availability to participate in the consensus, and blinded to the activities of the group A, was composed to assess the quality and clinical usefulness of the final draft of the structured report.

All panellist of group A analyzed literature papers on the main scientific databases, including Pubmed, Scopus, and Google Scholar, to assess papers on Mammography findings of Breast Cancer from December 2000 to December 2020. The full text of the selected studies was reviewed and helped the panelists to compose a first list of items of the reports, via emails and/or teleconferences.

SR was divided into 10 section: (a) Personal Data, (b) Setting, (c) Comparison with previous breast examination, (d) Anamnesis and clinical context; (e) Technique; (f) Radiation dose; (g) Parenchymal pattern; (h) Description of the finding; (i) Diagnostic categories and Report and (j) Conclusions. As a part of template we added a dedicated section of more relevant images.

### Delphi rounds

Preliminarily, each panellist autonomously provided to improving the draft of the SR by means of online meetings or mail exchanges. Subsequently, three Delphi rounds were performed [17].

During the first round, a Google Form survey was used to test the panellists’ agreement for the SR draft. Each section of the SR (i.e., Patient Clinical Data, Clinical Evaluation, Exam Technique, Report, Findings, and Conclusion) was tested by using a five-point Likert scale (1 = strongly disagree, 2 = slightly disagree, 3 = neither agree nor disagree 4 = slightly agree, 5 = strongly agree).

Afterward the second round, the final version of the structure report was generated on the dedicated RSNA website (radreport.org) by using T-Rex template in HTML format, in line with IHE (Integrating Healthcare Enterprise) and the MRRT (management of radiology report templates) profile, accessible as open-source software, with the technical support of Exprivia (Molfetta, Bari, Italy). These determine both the format of radiology report templates [by using both the version 5 of Hypertext Markup Language (HTML5)], and the transporting mechanism to request, get back, and stock these schedules [18]. The radiology report was structured by using a series of “codified queries” integrated in the T-Rex editor’s preselected sections [18].

In the third round of the Delphi process, the experts group B was asked to express their level of agreement, by using a five-point Likert scale, on the quality of reporting. In particular, the experts were asked to express the level of agreement on the following statements: 1) The structured report contains all the descriptive elements of a first diagnosis mammogram, 2) The structured report allows the diagnosis to be clearly expressed, 3) The structured report allows you to clearly indicate the patient’s management, 4) The structured report allows to reduce the reporting time compared to the descriptive one already used in clinical practice, 5) The structured report is easy for the radiologist to implement in clinical practice, 6) A training period for the radiologist is required to adopt the structured report.

## Statistical analysis

Each panellist answers were exported in Microsoft Excel document for data collection and statistical analysis.

Mean score, standard deviation, and the sum of scores were used as statistical descriptors of scores attributed by panellists for each section. A mean score of 3 was considered good while a score of 5 excellent.

The internal consistency of the panellist scores for each section was assessed and a quality analysis with the average inter-item correlation was performed using Cronbach’s alpha (Cα) correlation coefficient [22, 23]. Cα was determined after each round.

An alpha coefficient (α) > 0.9 was considered excellent, α > 0.8 good, α > 0.7 acceptable, α > 0.6 questionable, α > 0.5 poor, and α < 0.5 unacceptable. In the iterations an α of 0.8 was considered a reasonable goal for internal reliability.

The data analysis was performed using Statistic Toolbox of Matlab (The MathWorks, Inc., Natick, MA, USA).

## Results

### Structured report

The final SR version (Appendix 1) was built by including *n* = 2 items in Personal Data, *n* = 4 items in Setting, *n* = 2 items in Comparison with previous breast examination, *n* = 19 items in Anamnesis and clinical context; *n* = 10 items in Technique; *n* = 1 item in Radiation dose; *n* = 5 items Parenchymal pattern; *n* = 28 items in Description of the finding; *n* = 12 items in Diagnostic categories and Report and *n* = 1 item in Conclusions. Overall, 84 items composed the definitive version of SR.

The “Personal Data” section includes patient clinical information, as weight, height, BMI, waist circumference, pathologies as hyperglycemia, hypercholesterolemia, hypertriglyceridemia, arterial hypertension.

The “Setting” section clarifies the examination clinical setting, as organized screening assessment of recalls, diagnostic mammogram (spontaneous/opportunistic screening) in asymptomatic or symptomatic woman.

The “Comparison with previous breast examinations “section, when possible, includes data obtained from previous examinations, in order to compare current data with them.

The “Anamnesis and clinical context” section includes previous or familiarity to malignancies, risk factors, genetic panel as well as data on the presence of symptoms such as breast lump, axillary lump, nipple discharge, skin/nipple alterations, mastodynia or others.

The “Technique” section includes data on the type of exam performed, such as film screen mammography or digital mammography, as well as on the methodology used.

“Radiation dose” section includes data on the category of radiation exposure.

“Parenchymal pattern” section is based on ACR classification:

1. Almost entirely adipose tissue with sparse areas of fibroglandular tissue
2. Heterogeneously dense, with possible masking of small lesions
3. Homogeneously dense, with reduced sensitivity

The “Description of the finding” section includes data on lesion location, type of lesions (masses or not masses), size, shape, margins, density, the presence and the type of calcifications and associated clinical findings (such as skin retraction, skin thickening, nipple retraction, axillary adenopathy).

In the “Diagnostic categories and Report conclusions” section, the lesion is stratified in the different categories (negative, benign, probably benign finding, indeterminate lesion, finding highly suggestive of malignancy and known breast malignancy already demonstrated at histopathology), with consequent follow-up or diagnostic suggestion.

The “Conclusions” section is a free-text section, with radiological diagnosis.

### Consensus agreement

Table 1 reports single score and sum of scores of the 16 panellists for SR in the first round. One of the experts did not participate to the second round: Table 2 reports single score and sum of scores of panellists for SR in the second round.

**Table 1:** Single score and sum of scores of panellists for structured report (I round)

Panellist #	Personal data	1. Setting	2. Comparison with previous breast examinations	3. Anamnesis and diagnostic question	4. Informed consent to Mammography	5. Technique	6. Parenchymal pattern (ACR classification)	7.1. Location	7.2. Type of findings	7.3. Size	7.4. Associated changes	8. Diagnostic categories and Report conclusions	Sum of scores
1	5	5	5	5	5	5	5	5	4	5	5	5	59
2	3	5	5	5	5	5	5	5	5	5	5	5	58
3	4	5	5	5	5	5	5	5	4	5	4	5	57
4	5	5	5	5	4	5	5	5	5	5	5	5	59
5	5	5	5	5	5	5	5	5	5	5	5	5	60
6	2	3	5	4	2	3	5	3	5	5	2	5	44
7	5	3	5	5	5	5	5	5	5	5	5	5	58
8	5	5	5	5	5	5	5	5	5	5	5	5	60
9	5	5	5	5	3	5	5	5	5	5	5	5	58
10	5	4	5	4	4	4	5	5	5	5	5	5	56
11	5	5	5	5	5	3	4	5	5	5	5	5	57
12	4	4	5	5	5	5	4	5	5	5	5	5	57
13	4	4	5	4	2	4	4	4	5	5	5	4	50
14	3	5	5	3	5	2	4	5	4	5	5	3	49
15	3	5	4	5	5	5	5	5	5	5	5	4	56
16	4	5	5	5	4	5	5	5	5	5	5	5	58
Mean value	4.19	4.56	4.94	4.69	4.31	4.44	4.75	4.81	4.81	5.00	4.75	4.75	56.00
Standard deviation value	0.98	0.73	0.25	0.60	1.08	0.96	0.45	0.54	0.40	0.00	0.77	0.58	4.46

**Table 2:** Single score and sum of scores of panellists for structured report (II round)

Panellist #	Personal data	1. Setting	2. Comparison with previous breast examinations	3. Anamnesis and diagnostic question	5. Technique	6. Parenchymal pattern (ACR classification)	7.1. Location	7.2. Type of findings	7.3. Size	7.4. Associated changes	8. Diagnostic categories and Report conclusions	Sum of scores
1	5	5	5	5	5	5	5	5	5	5	5	55
2	5	5	5	5	4	5	5	5	5	4	5	53
3	5	5	5	5	5	5	5	5	5	5	5	55
4	2	5	5	5	5	5	5	5	5	5	5	52
5	5	5	5	5	5	5	5	5	5	5	4	54
6	5	5	5	2	5	5	5	2	5	5	5	49
7	5	5	5	5	5	5	5	5	5	5	5	55
8	5	5	5	5	4	5	5	5	5	5	5	54
9	4	5	5	5	5	5	5	5	5	5	5	54
10	5	5	5	5	5	5	5	5	5	5	5	55
11	5	5	3	5	5	5	5	5	5	5	5	53
12	5	5	5	5	5	5	5	5	4	5	5	54
13	5	5	5	5	5	5	5	5	5	5	5	55
14	5	5	5	5	5	5	5	5	5	5	4	54
15	5	5	5	5	5	5	5	5	5	5	5	55
Mean value	4.73	5.00	4.87	4.80	4.87	5.00	5.00	4.80	4.93	4.93	4.87	53.80
Standard deviation value	0.80	0.00	0.52	0.77	0.35	0.00	0.00	0.77	0.27	0.26	0.35	1.61

Both in the first and second round, as reported in Table 1 and 2, all parts had more than a good assessment. The overall mean score of the experts (n.16) and the sum of score for SR were 4.7 (range 2–5) and 896 (Table 1) in the first round. The overall mean score of the panellists (n.15) and the sum of score for SR were 4.9 (range 2–5) and 807 (Table 2) in the second round.

The overall mean score of the panellists in the second round was higher than the overall mean score of the first round with a lower standard deviation value.

The Cα correlation coefficient was 0.78 in the first round while was 0.82 in the second round for structured report.

Table 3 reports single score of panellists for structured report in the third round about the answers of 4 panellists about the SR quality evaluation. All questions received more than a good rating (≥ 3). The overall mean score of the experts (n.4) was 3.3 (range 2–5). The Cαcorrelation coefficient was 0.90 in this third round for structured report.

**Table 3:** Single score of panellists for structured quality evaluation (III round)

Panellist #	The structured report contains all the descriptive elements of a first diagnosis mammogram	The structured report allows the diagnosis to be clearly expressed	The structured report allows you to clearly indicate patient management	The structured report allows you to reduce the reporting time compared to the descriptive one you already use in clinical practice	The structured report is easy for the radiologist to implement in clinical practice	A training period for the radiologist is required to adopt the structured report
1	5	4	5	5	5	4
2	4	3	3	2	2	1
3	3	3	3	3	3	3
4	2	3	3	2	3	4
Mean value	3.50	3.25	3.50	3.00	3.25	3.00
Standard deviation value	1.29	0.50	1.00	1.41	1.26	1.41

## Discussion

In this study, a SR for x-ray mammography in the first diagnosis of breast cancer has been proposed and built with a multi-round Delphi modified consensus. An additional round has been introduced involved a group of experts, blinded to the activities of the group A, to evaluate the quality and the clinical usefulness of the final draft of the SR. Both in the first and second round all parts had more than a good assessment. The overall mean score of the panellists and the sum of score for SR were 4.7 and 896 in the first round. The overall mean score of the panellists and the sum of score for SR were 4.9 and 807 in the second round. The overall mean score of the experts in the second round was higher than the overall mean score of the first round with a lower standard deviation value to underline the higher agreement among the experts in the SR reached in this round. Regarding to the answers of panellists on the quality evaluation, all questions received more than a good rating (≥ 3). The overall mean score of the panellists was 3.3, although, regarding to the item that the structured report allows to reduce the reporting time compared to the descriptive one, two experts provided a score of 2 since they think that the time is similar in both cases. Regarding to the item that a training period for the radiologist to adopt the structured report should be required, an expert provided a score of 1 thinking that it is not necessary.

The Cronbach’s alpha (Cα) correlation coefficient was 0.90 in this third round for structured report.

With regard to “Personal data”, this section obtained mean and SD values slightly inferior to other sections, with a trend confirmed in both first and second “rounds”. In our opinion, it is due to the panellist idea that this meticulous process of data could slow down the normal work flow and was not considered to be easy to use. However, it is necessary to point out that all the sections are independent from each other and, therefore, this is an optional section which may not even be filled in, although it was conceived with the aim of creating databases. In fact, the possibility of collecting all these data allowed the creation of a large database, not only for epidemiological studies, but in the highest conception of radiology to lay the foundations for radiomics studies.

The present study provides the first mammography template established on standardized structure and lexicon, essential features for the observance to diagnostic-therapeutic proposal in order to reduce the uncertainty that could result from a non-standardized lexicon; it is authors opinion that the proposed structured report will enable a clear communication between radiologists and clinicians; of not the conclusion allow to express a definite diagnosis or a weighted differential diagnosis (DD) [24]. Several sections are included in the present template and, the evaluation of these allow to stratify the lesion in the different categories (negative, benign, probably benign finding, indeterminate lesion, finding highly suggestive of malignancy and known malignancy), with consequent follow-up or diagnostic suggestion. SR of mass lesions is based on the BI-RADS lexicon provided by the American College of Radiology [25]. The BI-RADS lexicon needs the understanding of radiologist to designate a final category. However, there is a significant inconstancy among radiologists for the assignment of BI-RADS categories due to the level of exercising site and the single radiologist [26, 27]. It is possible to reduce this variability to educating the readers in practice of the lexicon [26].

Several authors have reported that the use of a checklist may improve diagnostic accuracy [27–29]. The development of a SR to guide the assessment of the lesion should decrease variability among radiologists. Another key question is related to the presence of multiple lesions; however, radiologists usually described the lesion that is most essential to clinicians in defining the management of patients. Thus, identifying and extracting the index lesion is a critical clinical task [30, 31].

The present SR is built not only considering the categories suggested by the ACR and, therefore, should favor a correct evaluation of the lesion, but it is composed of different sections that allow the correlation of the radiological features with the clinical history. This radiology report is conceived to be rich in data that could potentially be pooled, analyzed, and correlated with patient outcomes, thereby informing future clinical and imaging guidelines. However, use of non-standardized lexicon should limit the effort of data collection across multiple institutions [32, 33].

Regarding to the “Technique” section, revealing the examination technique, not only within one’s own department, but also with departments of other centers, answers to a double reason. First, it permits the standardization of study protocols, and then, it permits to optimize the study protocols between the different centers. The protocol optimization should guide the quality progress through enhanced patient safety (e.g., radiation dose reduction), best practice, image quality and reduce medical error [34–40].

The benefits of SR over narrative report comprise standardized structure and lexicon, features mandatory for observance to diagnostic and therapeutic proposal and for admission in clinical trials. SR decreases the equivocality due to a non-uniform lexicon. Wide application of SR is essential to offer referring physicians the best quality of service and to researchers the best quality information in the setting of big data [38–54].

Despite the favorable results, there are several weaknesses which we should ponder. Firstly, the panelists were of the same country; the involvement of internationally specialists would permit a larger involvement and would spread the uniformity of the SR. Second, this study not assess the clinical effect of the SR on the managing of breast cancer patient. However, this study has the advantage of having been supported by a multidisciplinary team, where several experts have assessed the quality of the clinical impact.

## Conclusion

In this study, a structured reporting template for x-ray mammography in the first diagnosis of breast cancer, has been proposed and built with a multi-round Delphi modified consensus. An additional round has been introduced involved a group of experts, blinded to the activities of the group A, to assess the quality and clinical usefulness of the final draft of the structured report. Both in the first and second round all parts had more than a good assessment. A standardized approach with best practice guidelines will improve training in and the performance of assignment of BI-RADS assessment categories, and offer the base for quality assurance procedures within centers and across international borders.

## Data Availability

All data are reported in the manuscript.

## Template Mammography

Personal data.

● Weight, height, BMI, waist circumference.

● Pathologies.

o Absent.

o Present, specify:

1.: Setting
1.1.: Organized screening assessment of recalls
1.2.: Diagnostic mammogram (spontaneous/opportunistic screening) in asymptomatic woman
1.3.: Diagnostic mammogram in symptomatic woman
1.4.: Other
2.: Comparison with previous breast examinations
2.1.: Availability (exam and date; images and/or reports)
2.2.: Non-availability
3.: Anamnesis and clinical context
3.1.: Anamnesis
3.1.1.: Family and general clinical history
3.1.1.1.: Hormone replacing therapy
3.1.1.2.: Female lymphoma survivor who had chest radiation therapy
3.1.1.3.: Family history breast cancer ( who and age)
3.1.1.4.: Genes mutations
3.1.1.4.1.: BRCA1
3.1.1.4.2.: BRCA2
3.1.1.4.3.: TP53 (Li–Fraumeni syndrome)
3.1.1.4.4.: PTEN (Cowden syndrome)
3.1.1.4.5.: CDH1
3.1.1.4.6.: STK11 (Peutz–Jeghers syndrome)
3.1.1.4.7.: ATM
3.1.1.4.8.: CHEK2
3.1.1.4.9.: PALB2
3.1.1.5.: Others, specify
3.1.1.6.: Optional: breast cancer risk assessment
3.1.2.: Breast clinical history
3.1.2.1.: Previous percutaneous biopsy
3.1.2.2.: Previous surgery for benign lesions
3.1.2.3.: Previous additive mastoplasty
3.1.2.4.: Previous reductive mastoplasty
3.1.2.5.: Other
3.1.2.6.: If any, specify (location)
3.2.: Diagnostic question (if any)
3.2.1.: Asymptomatic subject
3.2.2.: Breast lump
3.2.3.: Axillary lump
3.2.4.: Nipple discharge
3.2.5.: Skin/nipple alterations
3.2.6.: mastodinia
3.2.7.: Symptoms of inflammation
3.2.8.: Other, specify
4.: Technique
4.1.: Bilateral
4.2.: One-sided
4.2.1.: Right
4.2.2.: Left
4.3.: Film screen mammography
4.4.: Digital mammography
4.4.1.: CR
4.4.2.: FFDM
4.5.: Tomosynthesis
4.6: Other
5.: Radiation dose: source DICOM data
6.: Parenchymal pattern
6.1.: ACR classification
6.1.1.: a) Almost entirely fatty tissue
6.1.2.: b) With sparse areas of fibroglandular tissue
6.1.3.: c) Heterogeneously dense, with possible masking of small lesions
6.1.4.: d) Homogeneously dense, with reduced sensitivity
6.2.: Automatic quantitative assessment
6.2.1.: Free text
7.: Description of the findings
7.1.: Location
7.1.1.: Laterality, quadrant (4 plus central-retroareolar), polar coordinates with respect to the nipple, depth and distance from the nipple
7.1.2.: Correspondence with clinical find (specify the clinical find)
7.1.2.1.: Yes
7.1.2.2: No
7.2.: Type of findings
7.2.1.: Masses
7.2.1.1.: Shape
7.2.1.1.1.: Oval
7.2.1.1.2.: Round
7.2.1.1.3.: Irregular
7.2.1.2.: Margins
7.2.1.2.1.: circumscribed
7.2.1.2.2.: Obscured
7.2.1.2.3.: Microlobulated
7.2.1.2.4.: Indistinct or ill defined
7.2.1.2.5.: Spiculate
7.2.1.3.: Density high-density
7.2.1.3.1.: Isodense or equal density
7.2.1.3.2.: Low-density
7.2.1.3.3.: Fat-containing
7.2.2.: Calcifications
7.2.2.1.: Morphology
7.2.2.1.1.: Typically benign (ring rim calcifications, round calcifications, other typically benign calcifications as skin, vascular, coarse or popcorn, dystrophic, calcium milk, and suture calcifications)
7.2.2.1.2.: Suspicious morphology (heterogeneously coarse, amorphous, finely pleomorphic, linear, or branched calcifications)
7.2.2.2.: Distribution pattern
7.2.2.2.1.: Grouped
7.2.2.2.2.: Segmental
7.2.2.2.3.: Regional
7.2.2.2.4.: Diffuse
7.2.2.2.5.: Linear
7.2.3.: Asymmetries
7.2.3.1.: Global increase
7.2.3.2.: Focal increase
7.2.4.: Architectural distortions
7.2.4.1.: With radiotransparent center
7.2.4.2.: With opacity center
7.3.: Size
7.3.1: The larger diameter in mm
7.3.2.: The comparison with the previous exam carried out with the date
7.4.: Associated changes
7.4.1.: Skin retraction, skin thickening, trabecular thickening, nipple retraction, axillary adenopathy. Of course, also architectural distortion and calcifications can be changes associated to other findings
8.: Diagnostic categories and Report conclusions
8.1.: Category 0. Additional imaging evaluation needed
8.1.1.: a) Use of spot compression, magnification or other special mammographic views, tomosynthesis, ultrasound, etc.
8.1.2.: b) When requesting previous images that were not available when evaluating the mammography
8.2.: Category 1-negative
8.2.1.: Subsequent mammography at 12 months
8.2.2.: Subsequent mammography at 24 months
8.3.: Category 2-benign
8.3.1.: Subsequent mammography at 12 months
8.3.2.: Subsequent mammography at 24 months
8.4.: Category 3- probably benign finding (< 2% malignancy)
8.4.1.: 6-months one-sided mammogram followed by bilateral diagnostic mammography at 12 months and 24 months
8.4.2.: Image-guided needle biopsy according to the radiologist’s choice, woman’s preference, or as resulting from the agreement with other clinicians
8.5.: Category 4 - indeterminate lesion
8.5.1.: Required further investigations
8.5.2.: Image-guided biopsy
8.6.: Category 5- finding highly suggestive of malignancy
8.6.1.: Image-guided biopsy
8.7.: Category 6- known breast malignancy already demonstrated at histopathology
9.: Conclusions (free text)
